# The pro-tumorigenic activity of p38γ overexpression in nasopharyngeal carcinoma

**DOI:** 10.1038/s41419-022-04637-8

**Published:** 2022-03-04

**Authors:** De-Pei Yin, Yu-Fan Zheng, Peng Sun, Ming-Yu Yao, Li-xiao Xie, Xun-Wu Dou, Ye Tian, Ji-Sheng Liu

**Affiliations:** 1 Department of Otorhinolaryngology Head and Neck Surgery, Children Hospital of Soochow University, Suzhou, China; 2 Jiangsu Key Laboratory of Neuropsychiatric Diseases and Institute of Neuroscience, Soochow University, Suzhou, China; 3 Department of Otorhinolaryngology Head and Neck Surgery, The First Affiliated Hospital of Soochow University, Suzhou, China; 4 Department of Radiotherapy and Oncology, The Second Affiliated Hospital of Soochow University, Suzhou, China

**Keywords:** Targeted therapies, Oncogenes

## Abstract

It is urgent to identify and validate biomarkers for early diagnosis and efficient treatment of nasopharyngeal carcinoma (NPC). Recent studies have proposed p38 gamma (p38γ) as a cyclin-dependent kinase (CDK)-like kinase that phosphorylates retinoblastoma (Rb) to promote cyclins expression and tumorigenesis. Here the Gene Expression Profiling Interactive Analysis (GEPIA) database and results from the local NPC tissues demonstrate that p38γ is significantly upregulated in NPC tissues, correlating with poor overall survival. Furthermore, *p38γ* mRNA and protein expression is elevated in established NPC cell lines (CNE-1 HONE-1 and CNE-2) and primary human NPC cells, but low expression detected in human nasal epithelial cells. In established and primary NPC cells, p38γ depletion, using the shRNA strategy or the CRISPR/Cas9 gene-editing method, largely inhibited cell growth, proliferation and migration, and induced significant apoptosis activation. Contrarily, ectopic p38γ overexpression exerted opposite activity and promoted NPC cell proliferation and migration. Retinoblastoma (Rb) phosphorylation and cyclin E1/A expression were decreased in NPC cells with p38γ silencing or knockout, but increased after p38γ overexpression. Moreover, mitochondrial subcellular p38γ localization was detected in NPC cells. Significantly, p38γ depletion disrupted mitochondrial functions, causing mitochondrial depolarization, reactive oxygen species production, oxidative injury and ATP depletion in NPC cells. In vivo, intratumoral injection of adeno-associated virus-packed p38γ shRNA potently inhibited primary human NPC xenograft growth in nude mice. In p38γ shRNA virus-injected NPC xenograft tissues, p38γ expression, Rb phosphorylation, cyclin E1/A expression and ATP levels were dramatically decreased. Taken together, we conclude that p38γ overexpression is required for NPC cell growth, acting as a promising therapeutic target of NPC.

## Introduction

Nasopharyngeal carcinoma (NPC) is a common nasopharynx epithelial malignancy with diverse etiopathogy and histopathology, causing ~65,000 cancer-related mortalities each year [1–3]. Its incidence can exceed 20 cases per 100,000 people in certain regions, including Southern China and Southeast Asia [4–6]. There are three subtypes of NPC, including squamous cell carcinoma, non-keratinizing carcinoma and undifferentiated carcinoma [4–6]. Epstein-Barr virus (EBV) infection is considered as a primary NPC risk factor [1–3]. Moreover, individuals that are exposed to cigarette smoking have an increased risk of developing NPC [1–3].

The current clinical treatments for NPC include surgery, platinum-based chemotherapy and radiotherapy [4–6]. The latter includes 3D conformal radiation therapy, intensity-modulated radiation therapy, particle beam therapy and brachytherapy [4–6]. It is estimated that 70–90% of NPC patients could respond well to radiotherapy (or in combination with chemotherapies) [1–3]. Yet, for the recurrent, metastatic and other advanced NPC patients, the progression-free survival (PFS) and overall survival (OS) are not satisfactory [1]. Treatments for these patients are largely limited to palliative systemic therapies [1, 4, 5]. It is therefore extremely important to identify and validate biomarkers for early diagnosis and efficient therapy of NPC [6].

In eukaryotic cells, p38 mitogen-activated protein kinases (MAPKs), p38α (MAPK14), p38β (MAPK11), p38γ (MAPK12), and p38δ (MAPK13), are responsive to various stimuli, including cytokines, different irradiation and heat shock [7, 8]. p38 MAPKs can regulate different cellular and physiological functions, including cell differentiation, apoptosis, immune regulation, cell growth, and autophagy, among others [9–11]. p38γ, also known as the extracellular signal-regulated kinase 6 (ERK6) or the stress-activated protein kinase 3 (SAPK3), can exert tumorigenic functions [12–18]. p38γ is shown to regulate K-Ras and other oncogenic proteins required for colorectal cancer (CRC) development [19]. Tomás-Loba et al. have discovered that p38γ can be a novel cyclin-dependent kinase (CDK)-like kinase, promoting cell-cycle progression and liver tumorigenesis [20]. p38γ phosphorylates retinoblastoma (Rb), the tumor suppressor protein, thereby increasing cyclin E1 and cyclin A expression and tumor cell growth [20]. p38γ depletion, using genetic strategies, potently inhibited tumor cell growth [20]. Nevertheless, the expression and potential functions of p38γ in NPC have not been studied. The results of this study will demonstrate the pro-tumorigenic activity of p38γ overexpression in NPC.

## Materials and methods

### Reagents

Cell culturing reagents, including fetal bovine serum (FBS), RPMI-1640, DMEM and antibiotics were provide by Gibco Co. (Carlsbad, CA). The Cell counting kit −8 (CCK-8) was purchased from Dojindo (Kumamoto, Japan). The fluorescence dyes, including CellROX, DAPI (4’,6-diamidino-2-phenylindole), TUNEL (Terminal deoxynucleotidyl transferase dUTP nick end labeling) and JC-1, were obtained from Invitrogen Thermo-Fisher Scientific (Suzhou, China). Antibodies for cyclin E1 (#4129), cyclin-dependent kinase 2 (CDK2, #2546), VDAC1 (#4661), α-Tubulin (#2144), Lamin B1 (#13435), β-Tubulin (#2146), cyclin A (#4656) and Rb antibody sampler kit (#9969) were purchased from Cell Signaling Technologies (Beverly, MA). Antibodies for PFKFB3 (6-phosphofructo-2-kinase/fructose-2,6-biphosphatase 3, #13123), p-PFKFB3 and glucose transporter 2 (GLUT2) were described previously [12]. The p38γ specific pharmacological inhibitor pirfenidone (PFD) was obtained from Sigma (Shanghai, China).

### Cell culture

The immortalized NPC cell lines, including CNE-1, HONE-1 and CNE-2, were purchased from the Cell Bank of Shanghai Institute of Biological Science, CAS (Shanghai, China). Cells were cultivated in RPMI-1640/DMEM supplemented with 8–10% FBS and antibiotics, at 37 °C in a humidified 5% CO_2_ incubator. The primary human NPC cells that were derived from one primary NPC patient, namely pNPC-1, as well as the primary human nasal epithelial cells (HNEpC) that were derived from two donors (pHNEpC-1 and pHNEpC-2), were provided by Dr. Chen at Jiangsu University [21]. The protocols of using human cells were approved by the Ethic Committee of Soochow University, in according with the principles of Declaration of Helsinki.

### Human tissues

NPC tumor tissues and the matched adjacent nasopharynx epithelial tissues were from a set of fifteen (15) primary NPC patients. The patients were administrated at authors’ institutions, provided written-informed consents and received no prior therapies before surgeries. Tissues were stored in liquid nitrogen. The protocols were approved by the Ethics Committee of Soochow University, in according to the principles of Declaration of Helsinki.

### p38γ silencing or overexpression

The two non-overlapping shRNAs targeting p38γ (p38γ-shRNA-s1 and p38γ-shRNA-s2, from Dr. Shi [22]) and a negative control with the scrambled non-sense sequence (shC) were constructed into GV248 lentiviral vectors provided by Shanghai Genechem Co. (Shanghai, China). The full-length p38γ cDNA sequence (also from Dr. Shi [22]) was sub-cloned into the GV248 lentiviral vector (Genechem Co.). The lentivirus was produced by transfecting HEK293T cells with the plasmids using a lentivirus packaging mix (Genechem Co.). The virus was enriched and filtered. Cells were then infected with the virus and selected with puromycin for 96 h. Expression of p38γ in the stable cells was verified by qRT-PCR and Western blotting assays. For in vivo studies the p38γ-shRNA-s1 sequence was inserted into the adeno-associated virus (aav) construct, aav9 (Genechem, Shanghai, China).

### p38γ knockout (KO)

A CRISPR/Cas9 PX458-GFP construct containing p38γ small guide RNA (sgRNA) was provided by Dr. Shi [22]. NPC cells were placed into six-well plates and transfected with Cas9-expressing construct (Genechem, Shanghai, China). The stable Cas9-expressing cells were established after puromycin selection. Cells were then transfected with the CRISPR/Cas9-p38γ-KO construct. The transfected cells were distributed into 96-well plates for 96 h, subject to p38γ KO screening using qPCR and Western blotting assays. At last, the single stable monoclonal p38γ knockout (p38γ-KO) NPC cells were established.

### Gene detection

Detailed protocols for Western blotting, quantitative reverse transcription-polymerase chain reaction (qRT-PCR), co-immunoprecipitation (Co-IP) were described previously [23–25]. The primers of this study were provided by Dr. Shi at Soochow University [22]. The isolation of mitochondria through the high-speed centrifugation was through the Pierce kit (Pierce Biotechnology, Rockford, IL) according to the protocols attached. The uncropped blotting images were presented in Supplementary Fig. S1.

### Functional assays

Transwell migration and invasion assays, CCK-8 viability assay, clonogenic assay and 5-ethynyl-20-deoxyuridine (EdU) proliferation assays as well as nuclear TUNEL staining and Annexin V FACS apoptosis assays were described in detail in other studies [23, 26, 27].

### Reactive oxygen species (ROS) detection

Production of ROS was measured by a CellROX probe [28]. Cells with the designated genetic modifications were seeded into 96-well plates and were stained with CellROX Deep Red (10 µM) for 30 min at 37 °C. Cells were then washed twice. CellROX fluorescence was measured at 625 nm under a Fluoroskan Ascent FL microplate reader. The fluorescence was also photographed under an Olympus fluorescence microscope (Olympus, Tokyo, Japan).

### Measuring mitochondrial membrane potential

Cells with the designated genetic modifications were seeded into 24-well plates. Afterward, cells were incubated in total darkness with 10 µM JC-1 (Invitrogen) for 45 min at room temperature [29]. JC-1 green monomer intensity was measured under a Fluoroskan Ascent FL microplate reader. JC-1 green monomers and red dimers (J-aggregates) were photographed as well under an Olympus fluorescence microscope (Olympus, Tokyo, Japan).

### Caspase activity

The activities of caspase-3 and caspase-7 were examined by an Apo-ONE Homogeneous caspase 3/7 assay kit (Promega Corporation, Madison, WI) according to the manufacturer’s protocols.

### Histone DNA ELISA and ssDNA ELISA

Cells with the designated treatments were plated into the 96-well plates at a density of 3 × 10^3^ cells/well. After culturing for applied time periods, the Histone-bound DNA contents and single-strand DNA (ssDNA) contents were analyzed by the corresponding ELISA kits (Roche, Shanghai, China). The ELISA OD at 450 nm in each well was recorded.

### ATP contents

NPC cells with the applied genetic treatments were placed into 12-well plates at 1 × 10^5^ cells per well and cultured for applied time periods. An ATP assay kit (Biyuntian, Wuxi, China) was utilized to quantify ATP contents according to the attached protocols.

### Rb mutation

As described previously [30], the non-phosphorylated mutant human Rb (“Rb-mut”) was generated by changing 15 Ser/Thr sites to Ala, with Ser567 left unaltered, and a HA tag placed on the N-terminus. The Rb-mut construct was generated by Genechem (Shanghai, China) and was transfected to transduced to p38γ-overexpressed CNE-1 cells (OE-p38γ-L1) through Lipofectamine 3000 (24 h per round for three rounds).

### Xenograft assay

The nude mice were half male half female, 4–5 week old and 18.2–19.2 g in weight. Mice were provided by Shanghai SLAC Laboratory Animal Center (Shanghai, China). Mice were housed under the Guide for the Care and Use of Laboratory Animals (NIH). The primary human NPC cells, pNPC-1, were subcutaneously (*s.c*.) injected to the flanks of the nude mice. Five million cells in 100 µL of DMEM/Matrigel (no serum) were inoculated. The volume of each xenograft was close to 100 mm^3^ within three weeks (labeled at “Day-0”). The xenograft-bearing mice were then randomly assigned into two groups (ten mice per group), receiving intratumoral injection of designated adeno-associated virus (aav)-packed shRNA. The tumor volume was determined using the described formula [31]. The protocols were approved by the Ethics Committee and Institute Animal Ethics Review Board of Soochow University.

### Statistical analyses

Data were with normal distribution and were presented as mean ± standard deviation (SD). Statistical analyses were carried out using the Student’s *t* test (Excel 2007) for comparisons between two groups, or one-way ANOVA plus a Scheffe’ and Tukey Test (SPSS 23.0) for comparisons between multiple groups. *P* < 0.05 was considered to indicate a significant difference. In vitro experiments were repeated five times.

## Results

### p38γ is overexpressed in NPC

**Fig. 1:**
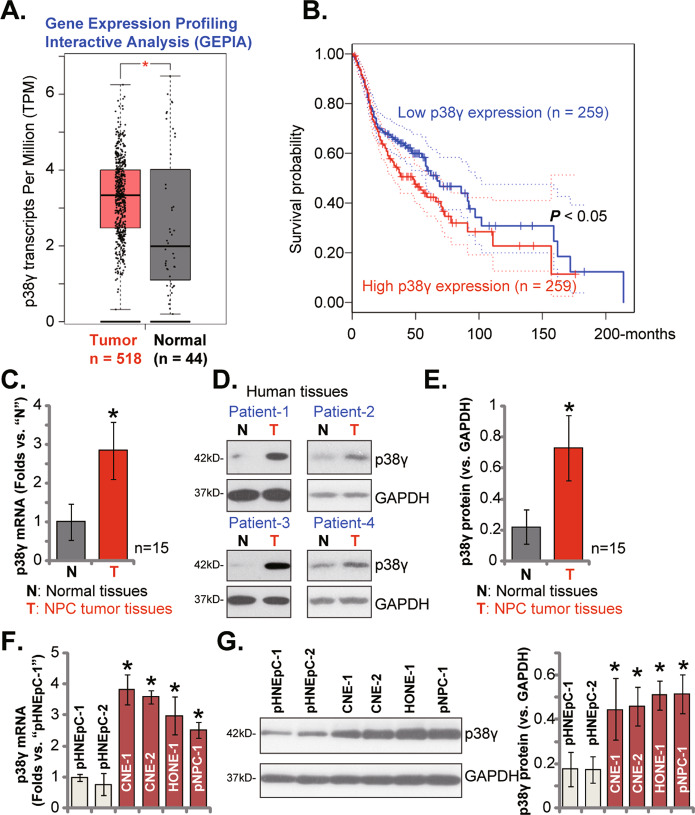
p38γ is overexpressed in NPC. The Gene Expression Profiling Interactive Analysis (GEPIA) database shows *p38γ* mRNA transcripts in NPC tumor tissues (“Tumor”, *n* = 518) and the normal nasopharynx epithelial tissues (“Normal”, *n* = 44) (**A**). The Kaplan–Meier Survival analyses of the *p38γ*-low (*n* = 259) and the *p38γ*-high (*n* = 259) NPC patients (**B**). Expression of *p38γ* mRNA (**C**) and protein (**D** and **E**) in local NPC tumor tissues (“T”, *n* = 15) and the matched adjacent nasopharynx epithelial tissues (“N”, *n* = 15) was shown, with results quantified. Expression of *p38γ* mRNA (**F**) and protein (**G**) in the listed NPC cells and the primary human nasal epithelial cells (pHNEpC-1 and pHNEpC-2) was tested as well. *P < 0.05 *vs*. “Normal”/“N” tissues or pHNEpC-1 cells.

The Gene Expression Profiling Interactive Analysis (GEPIA) database was first consulted to test *p38γ* mRNA transcripts in NPC. Results showed that *p38γ*transcripts in NPC tumor tissues (“Tumor”, *n* = 518) were significantly higher than those in the normal nasopharynx epithelial tissues (“Normal”, *n* = 44) (Fig. 1A). Kaplan-Meier survival analyses, Fig. 1B, found that high p38γ expression in NPC patients correlated with poor survival.

To verify the bioinformatics results, we tested p38γ expression in local NPC tissues. A total of fifteen (*n* = 15) primary NPC patients were enrolled and fresh tissue specimens were obtained. The qRT-PCR assay results, Fig. 1C, showed that *p38γ* mRNA levels in NPC tumor tissues (“T”) were significantly higher than those in the matched adjacent nasopharynx epithelial tissues (“N”). Testing p38γ protein expression, using Western blotting assays, further confirmed p38γ protein upregulation in NPC tumor tissues of four representative patients (“Patient #1/#2/#3/#4”) (Fig. 1D). The blotting data of all human tissues were combined. As shown, the p38γ protein expression in NPC tumor tissues was significantly higher than that in the normal nasopharynx epithelial tissues (Fig. 1E).

Next, the p38γ expression in different NPC cells was tested. As shown, *p38γ* mRNA (Fig. 1F) and protein (Fig. 1G) expression levels were upregulated in immortalized NPC cell lines (CNE-1, HONE-1 and CNE-2) as well as in the primary human NPC cells (“ pNPC-1”). Conversely, the low expression was detected in the primary human nasal epithelial cells (HNEpC) that were derived from two primary donors, pHNEpC-1 and pHNEpC-2 (Fig. 1F, G). These results show that p38γ is overexpressed in NPC.

### p38γ shRNA inhibits NPC cell progression in vitro

**Fig. 2:**
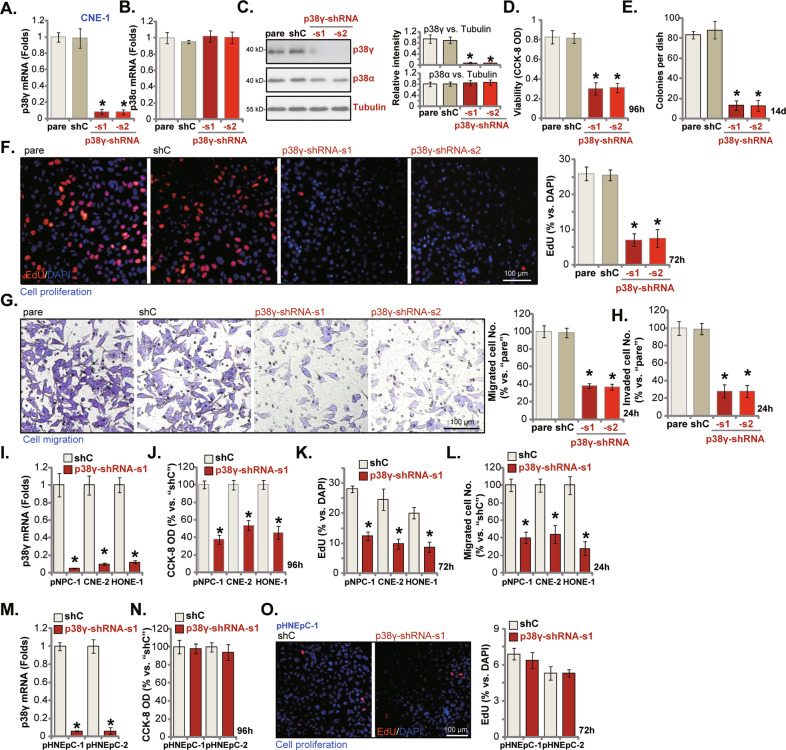
p38γ shRNA inhibits NPC cell progression in vitro. The immortalized NPC cell lines (CNE-1, HONE-1 and CNE-2) (**A**–**L**), the primary human NPC cells (“pNPC-1”, **I**–**L**), or the primary human nasal epithelial cells (pHNEpC-1 and pHNEpC-2, derived from two donors) (**M**–**O**) were infected with lentivirus-encoded shRNA (p38γ-shRNA-s1, p38γ-shRNA-s2 or the the scramble control shRNA/shC), with stable cells established after puromycin selection, expression of listed genes was tested by qRT-PCR (**A**, **B**, **I** and **M**) and Western blotting (**C**) assays. Cells were further cultured for designated periods, cell viability (by recording CCK-8 intensities, **D**, **J** and **N**), colony formation (**E**) and cell proliferation (by testing the EdU-positive nuclei ratios, **F**, **K** and **O**) as well as cell migration (“Transwell” assays, **G** and **L**) and invasion (“Matrigel Transwell” assays, **H**) were tested by the indicated assays. “pare” stands for the parental control cells. *P < 0.05 *vs*. “shC” cells. Scale Bar = 100 μm (**F**, **G** and **O**).

To silence p38γ, two different lentiviral shRNAs targeting non-overlapping sequences of *p38γ*, p38γ-shRNA-s1 and p38γ-shRNA-s2 (from Dr. Shi [22]) were utilized. CNE-1 cells were infected with the lentiviral p38γ shRNA, and stable cells established after puromycin selection. Examining mRNA expression, using qRT-PCR assays, demonstrated that the *p38γ* mRNA levels decreased over 90% in stable CNE-1 cells expressing the two p38γ-shRNAs (Fig. 2A). The *p38α* mRNA expression was however unaffected (Fig. 2B). Western blotting assay results, Fig. 2C, confirmed robust p38γ protein silencing in p38γ-shRNA-expressing stable CNE-1 cells, where p38α protein expression was unaffected (Fig. 2C). As expected, the scramble control shRNA, shC, did not alter p38α and p38γ expression in CNE-1 cells (Fig. 2A–C).

To test whether p38γ silencing could affect the functions of NPC cells, CCK-8 assays were performed. Results showed that CCK-8 viability was dramatically decreased in stable CNE-1 cells expressing p38γ-shRNA (Fig. 2D). Moreover, shRNA-induced silencing of p38γ potently decreased the number of viable CNE-1 cell colonies (Fig. 2E), further supporting the anti-survival activity by p38γ-shRNA. p38γ silencing induced significant anti-proliferative activity in CNE-1 cells, and the EdU-positive nuclei ratio was dramatically decreased in p38γ-shRNA-expressing cells (Fig. 2F). Further, p38γ-shRNA suppressed CNE-1 cell in vitro migration and invasion, which were examined through “Transwell” (Fig. 2G) and “Matrigel Transwell” (Fig. 2H) assays, respectively. Unsurprisingly, the control shC treatment failed to affect CNE-1 cell functions (Fig. 2D–H).

We also examined the potential function of p38γ in other NPC cells. The primary human NPC cells, pNPC-1, as well as two other established lines, HONE-1 and CNE-2, were cultured and infected with p38γ-shRNA-s1 lentivirus. Stable cells were again established by using puromycin selection medium. Figure 2I confirmed robust p38γ mRNA downregulation in the p38γ-shRNA-s1-expressing cells. In these NPC cells, p38γ shRNA potently inhibited cell viability, causing CCK-8 OD reduction (Fig. 2J). Moreover, it significantly decreased the EdU-positive nuclei ratio (Fig. 2K) and the number of migrated NPC cells (Fig. 2L).

To study the potential function of p38γ in non-cancerous epithelial cells, the primary human nasal epithelial cells (HNEpC)-derived from two primary donors, pHNEpC-1 and pHNEpC-2, were cultured and infected with the p38γ-shRNA-s1 lentivirus. In the stable cells, depleted *p38γ* mRNA expression was detected (Fig. 2M). Significantly, shRNA-induced silencing of p38γ did not significantly inhibit the viability (CCK-8 OD, Fig. 2N) and proliferation (by measuring the EdU-positive nuclei ratio, Fig. 2O) in the nasal epithelial cells (Fig. 2O). These results supported a cancer cell-specific effect of p38γ shRNA.

### Apoptosis activation by p38γ shRNA in NPC cells

**Fig. 3:**
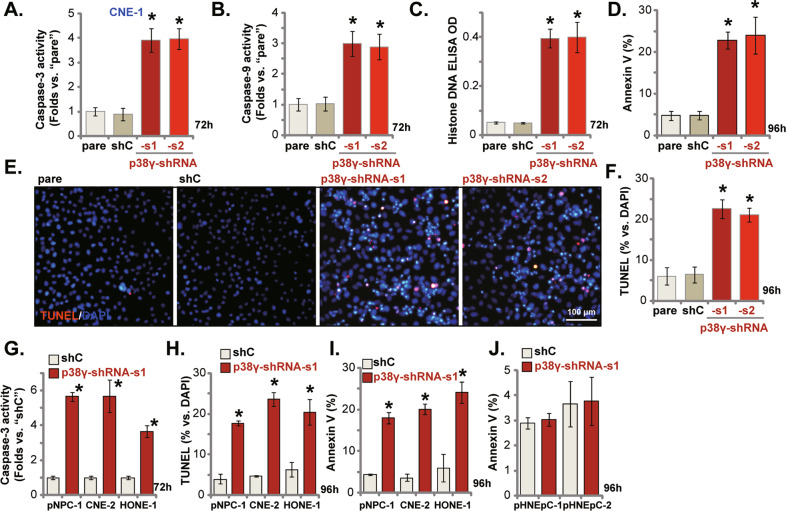
Apoptosis activation by p38γ shRNA in NPC cells. The immortalized NPC cell lines (CNE-1, HONE-1 and CNE-2) (**A**–**I**), the primary human NPC cells (pNPC-1, **G**–**I**), or the primary human nasal epithelial cells (pHNEpC-1 and pHNEpC-2) (**J**) were infected with lentiviral shRNA (p38γ-shRNA-s1, p38γ-shRNA-s2 or the scramble control shRNA/shC), with stable cells established after puromycin selection; Cells were further cultured for designated periods, caspase-3/-9 activation was examined (**A**, **B** and **G**), and histone-bound DNA contents examined by the ELISA assays (**C**); Cell apoptosis was tested by Annexin V FACS (**D**, **I** and **J**) nuclear TUNEL staining (**E**, **F** and **H**) and assays, with results quantified. “pare” stands for the parental control cells. *P < 0.05 *vs*. “shC” cells. Scale Bar = 100 μm (**E**).

Recent studies have implied that p38γ silencing could provoke apoptosis activation in cancer cells [13, 14, 22]. Here in the stable CNE-1 cells expressing p38γ-shRNA-s1/s2, the caspase-3 activity (Fig. 3A) and the caspase-9 activity (Fig. 3B) were both significantly increased. The histone-bound DNA contents were increased in p38γ-silenced CNE-1 cells (Fig. 3C). p38γ silencing induced significant apoptosis activation in CNE-1 cells, and the number of Annexin V-positive CNE-1 cells (Fig. 3D) and the TUNEL-positive nuclei ratio (Fig. 3E, F) were significantly increased in p38γ-shRNA-expressing cells. shC treatment failed to provoke significant caspase and apoptosis activation in CNE-1 cells (Fig. 3A–F). Similarly in the primary human NPC cells (pNPC-1) and other immortalized cell lines (HONE-1 and CNE-2), p38γ-shRNA-s1-induced silencing of p38γ provoked caspase-3 activation (Fig. 3G). The quantified nuclear TUNEL staining assay results in Fig. 3H and the Annexin V FACS assay results in Fig. 3I further confirmed apoptosis activation by p38γ shRNA in the primary and immortalized NPC cells. Conversely, the same p38γ-shRNA-s1 treatment failed to induce significant apoptosis activation in the primary nasal epithelial cells, pHNEpC-1 and pHNEpC-2 (Fig. 3J).

### The anti-NPC cell activity by p38γ KO

**Fig. 4:**
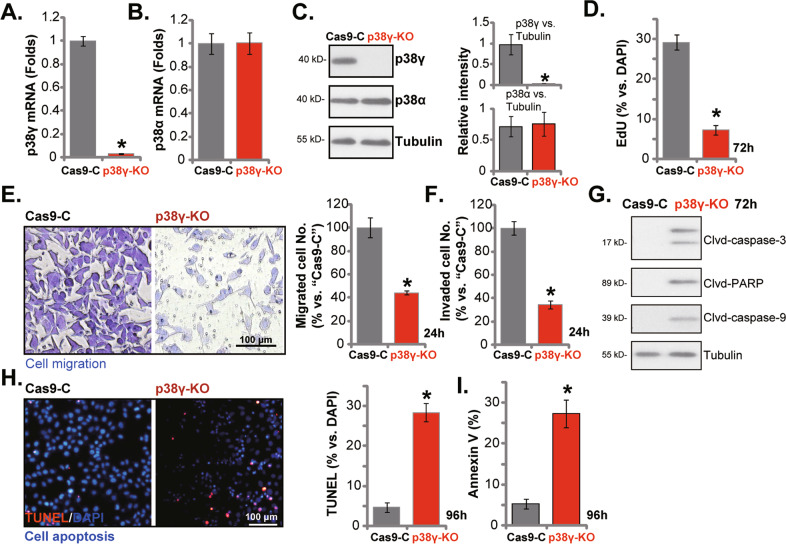
The anti-NPC cell activity by p38γ KO. Stable CNE-1 cells expressing a lenti-CRISPR/Cas9-p38γ-KO construct (“p38γ-KO”) or the lenti-CRISPR/Cas9-control construct (“Cas9-C”) were established, expression of listed genes was tested by qRT-PCR (**A** and **B**) and Western blotting (**C**) assays. Cells were further cultured for designated periods, cell proliferation (by recording the EdU-positive nuclei ratios, **D**), cell migration (“Transwell” assays, **E**) and invasion (“Matrigel Transwell” assays, **F**), as well as caspase-PARP cleavages (**G**) were tested. Cell apoptosis was tested by nuclear TUNEL staining (**H**) and Annexin V FACS (**I**) assays, and results quantified. *P < 0.05 *vs*. “Cas9-C” cells. Scale Bar = 100 μm (**E**, **H**).

To further support the role of p38γ in NPC cells, the CRISPR/Cas9 method was utilized to knockout p38γ. As described, a lenti-CRISPR/Cas9-p38γ-KO construct was transduced to Cas9-expressing stable CNE-1 cells. Following p38γ KO screening, the single stable p38γ knockout CNE-1 cells were established: p38γ-KO cells. As compared to cells with the lenti-CRISPR/Cas9-control construct (Cas9-C), the *p38γ* mRNA expression was completely depleted in the p38γ-KO cells (Fig. 4A). *p38α* mRNA expression was unchanged (Fig. 4B). Depletion of p38γ protein, but not p38α protein, was observed in the p38γ-KO CNE-1 cells (Fig. 4C). CRISPR/Cas9-induced p38γ KO largely inhibited CNE-1 cell proliferation, which was evidenced by significantly decreased EdU-positive nuclei ratio (Fig. 4D). p38γ KO also inhibited CNE-1 cell in vitro migration (Fig. 4E) and invasion (Fig. 4F). Western blotting assay results showed that in the p38γ-KO cells CNE-1 cells, levels of cleaved-caspae-3, cleaved-caspae-9 and cleaved-PARP1 were significantly increased (Fig. 4G). Moreover, p38γ KO induced significant apoptosis activation in CNE-1 cells, which was evidenced by the increased TUNEL-positive nuclei ratio (Fig. 4H) and Annexin V-positive staining (Fig. 4I). These results showed that CRISPR/Cas9-induced p38γ KO induced significant anti-cancer activity in CNE-1 cells.

### The cancer-promoting activity by p38γ overexpression in NPC cells

**Fig. 5:**
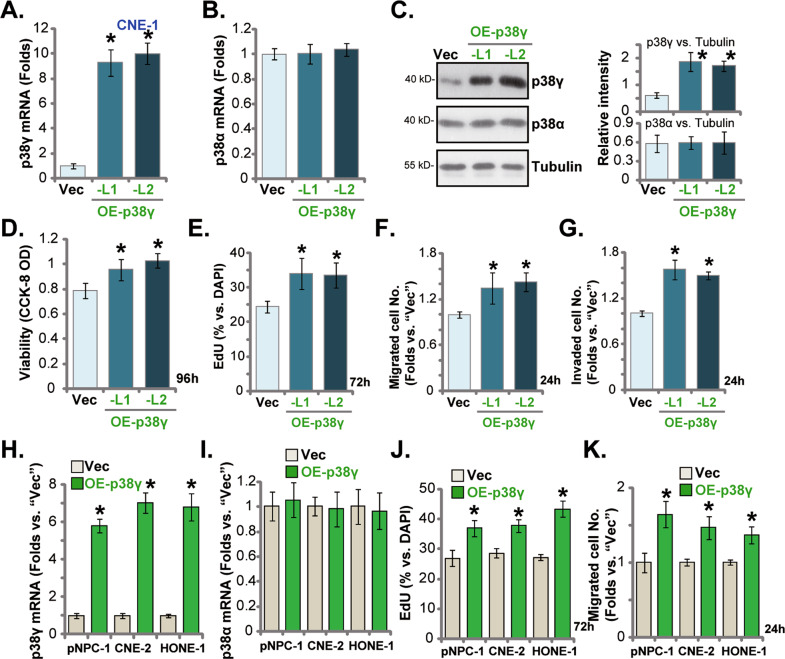
The cancer-promoting activity by p38γ overexpression in NPC cells. The immortalized NPC cell lines (CNE-1, HONE-1 and CNE-2) (**A**–**K**) or the primary human NPC cells (pNPC-1, **H**–**K**) were infected with the lentivirus encoding the p38γ-expressing construct (“OE-p38γ”) or the lentivirus with the empty vector (“Vec”), with stable cells established after puromycin selection; Expression of listed genes was tested by qRT-PCR (**A**, **B**, **H** and **I**) and Western blotting (**C**) assays. Cells were further cultured for designated periods, cell viability (by recording CCK-8 OD, **D**) and cell proliferation (by recording the EdU-positive nuclei ratio, **E** and **J**) as well as cell migration (“Transwell” assays, **F** and **K**) and invasion (“Matrigel Transwell” assays, **G**) were tested by the indicated assays, with results quantified. *P < 0.05 *vs*. “Vec” cells.

The results showed that shRNA-induced p38γsilencing or CRISPR/Cas9-induced p38γ KO exerted significant anti-cancer activity in NPC cells, we therefore tested whether p38γ overexpression could exert opposite activity. The lentivirus encoding the p38γ-expressing construct was added to CNE-1 cells. Following selection by puromycin, two stable cell lines, OE-p38γ-L1 and OE-p38γ-L2, were established. As compared to the vector control cells (“Vec”), expression of the *p38γ* mRNA (Fig. 5A), but not *p38α* mRNA (Fig. 5B), was significantly increased in the OE-p38γ CNE-1 cells. p38γ protein expression was significantly elevated as well in OE-p38γ-L1/L2 CNE-1 cells (Fig. 5C), where p38α protein expression was unchanged (Fig. 5C). The CCK-8 OD was augmented in p38γ-overexpressed CNE-1 cells (Fig. 5D). In addition, the EdU-positive nuclei ratio was increased in the OE-p38γ CNE-1 cells (Fig. 5E), indicating that ectopic p38γ overexpression-promoted CNE-1 cell proliferation. The numbers of migrated and invaded CNE-1 cells were enhanced by ectopic p38γ overexpression (Fig. 5F, G).

To the primary human NPC cells (pNPC-1) and other immortalized cells (HONE-1 and CNE-2), the p38γ-expressing lentivirus was added, and stable cells established after puromycin selection: OE-p38γ. In the OE-p38γ NPC cells, the *p38γ* mRNA levels were significantly increased (Fig. 5H), but *p38α* mRNA levels were unchanged (Fig. 5I). In line with the results of CNE-1 cells, ectopic overexpression of p38γ in the NPC cells augmented cell proliferation (increased EdU-positive nuclei ratio, Fig. 5J) and the number of the migrated cells (Fig. 5K).

### p38γ silencing decreases Rb phosphorylation, cyclin E1/A expression and disrupts mitochondrial functions in NPC cells

**Fig. 6:**
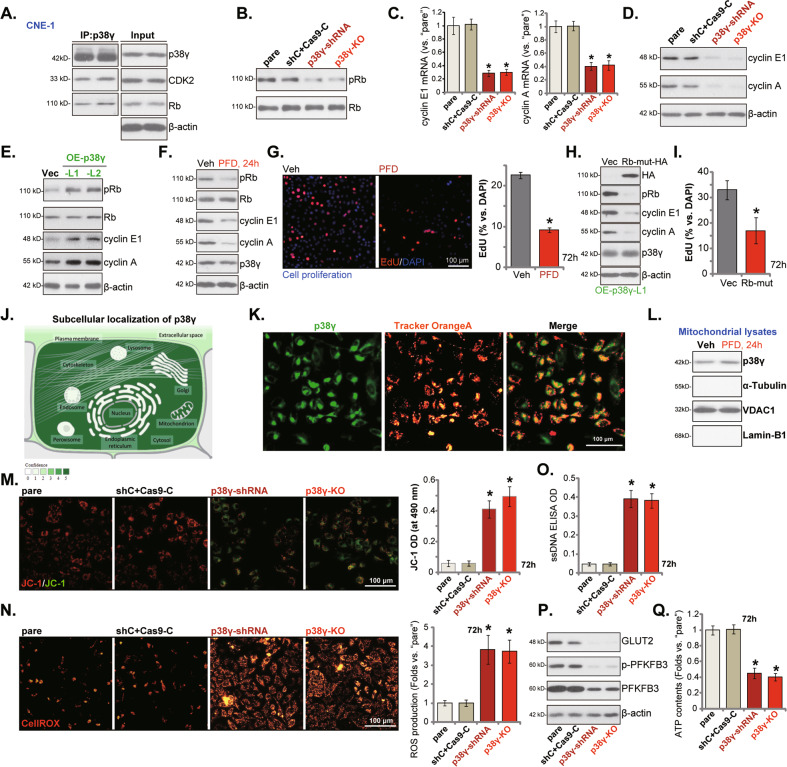
p38γ silencing decreases Rb phosphorylation, cyclin E1/A expression and disrupts mitochondrial functions in NPC cells. Co-immunoprecipitation (Co-IP) assay tested the association between p38γ, CDK2 and Rb (**A**) in CNE-1 cells. Their expression was presented in “Input” (**A**). The stable CNE-1 cells expressing the p38γ-shRNA-s1 (“p38γ-shRNA”), the lenti-CRISPR/Cas9-p38γ-KO construct (“p38γ-KO”), or the scramble control shRNA plus the lenti-CRISPR/Cas9-control construct (“shC+Cas9-C”) were established, expression of listed proteins and mRNAs was shown (**B**–**D** and **P**); Cells were further cultured for applied time periods, mitochondrial depolarization (by measuring JC-1 green monomer intensity, **M**), ROS contents (by measuring CellROX intensity, **N**), ssDNA contents (**O**) and ATP contents (**Q**) were tested. CNE-1 cells expressing the lentiviral p38γ-expressing construct (“OE-p38γ-L1/L2”) or the empty vector (“Vec”) were established, expression of listed proteins was shown (**E**). CNE-1 cells were treated with the p38γ specific pharmacological inhibitor pirfenidone (PFD, 0.5 mg/mL) or the vehicle control (0.1% DMSO, “Veh”) for indicated time periods, expression of listed proteins in total cell lysates (**F**) and mitochondrial fraction lysates (**L**) were shown; Cell proliferation was tested by EdU staining assays (**G**). OE-p38γ-L1 CNE-1 cells were further transduced with A non-phosphorylated Rb mutant construct (“Rb-mut”) or the empty vector (“Vec”), expression of listed proteins was shown (**H**); Cells were further cultured for 72 h and cell proliferation was tested by measuring EdU-positive nuclei ratio (**I**). The Compartments Database shows the subcellular localization of p38γ protein (**J**). The confocal fluorescence images showed p38γ protein (in green fluorescence) and MitoTracker Orange (in orange fluorescence) co-localization in CNE-1 cells (**K**). “pare” stands for the parental control cells. *P < 0.05 *vs*. “pare”/“Vec” cells. Scale Bar = 100 μm (**G**, **K**, **M** and **O**).

Studies have shown that p38γ can associate with CDK2 (cyclin-dependent kinase 2) and retinoblastoma (Rb), inducing Rb phosphorylation at multiple residues, including Ser807, Ser811 and Ser780 [13, 15, 20]. We demonstrated that Rb immunoprecipitated with p38γ and CDK2 in CNE-1 cells (Fig. 6A). Figure 6B demonstrated that Rb phosphorylation (Ser 807/811) was significantly decreased in stable CNE-1 cells expressing the p38γ-shRNA-s1 (“p38γ-shRNA”) or the lenti-CRISPR/Cas9-p38γ-KO construct (“p38γ-KO”). Moreover, mRNA (Fig. 6C) and protein (Fig. 6D) expression of cyclin E1 and cyclin A were significantly decreased in p38γ-silenced or p38γ-KO CNE-1 cells. The scramble control shRNA plus the lenti-CRISPR/Cas9-control construct (“shC+Cas9-C”) treatment, unsurprisingly, did not alter Rb phosphorylation as well as cyclin E1 and cyclin A expression (Fig. 6B–D). On the contrast, in the OE-p38γ CNE-1 cells (OE-p38γ-L1/L2, see Fig. 5), Rb phosphorylation was significantly increased (Fig. 6E). Cyclin E1 and cyclin A protein levels were augmented (Fig. 6E). In addition, the p38γ specific pharmacological inhibitor pirfenidone (PFD) inhibited Rb phosphorylation (Fig. 6F), cyclins expression (Fig. 6F) and impeded CNE-1 cell proliferation (evidenced by decreased EdU-positive nuclei ratio, Fig. 6G). These results implied that p38γ is essential for Rb phosphorylation and cyclin E1/A expression in NPC cells.

Next, a non-phosphorylated mutant Rb (Rb-mut) [30] was transduced to p38γ-overexpressed CNE-1 cells (OE-p38γ-L1). As shown Rb-mut (HA-tag) almost blocked Rb phosphorylation and decreased cyclin E1/A expression in OE-p38γ-L1 cells (Fig. 6H). Notably, p38γ overexpression-promoted CNE-1 cell proliferation (by testing EdU-positive nuclei ratio) was inhibited by Rb-mut (Fig. 6I).

Besides locating in cell nuclei and cytosol, results from the Compartments Database (https://compartments.jensenlab.org) implied that p38γ can also locate at the mitochondrion (Fig. 6J). We therefore analyzed whether p38γ depletion could affect mitochondrial functions in OS cells. The fluorescence confocal microscope results found that the p38γ protein (in green fluorescence) indeed localized to the mitochondria (labeled with MitoTracker Orange, in orange fluorescence) in CNE-1 cells (Fig. 6K). Furthermore, an examination of mitochondrial lysates isolated from CNE-1 cells demonstrated that p38γ was indeed enriched in the mitochondrial fraction (Fig. 6L), as indicated by VDAC1 (voltage-dependent anion-selective channel 1), the mitochondrial marker protein (Fig. 6L). Lamin-B1 is the nuclear marker protein and α-Tubulin is the cytosol marker protein (Fig. 6L). Significantly, the p38γ inhibitor PFD failed to affect p38γ mitochondrial enrichment in CNE-1 cells (Fig. 6L).

Significantly, p38γ silencing or KO disrupted mitochondrial functions, causing mitochondrial membrane potential reduction and mitochondrial depolarization. The latter was evidenced by JC-1 green monomer accumulation (Fig. 6M). Furthermore, the CellROX intensity was significantly increased in p38γ-shRNA cells and p38γ-KO CNE-1 cells, indicating ROS production and oxidative injury (Fig. 6N). In addition, the ssDNA contents were increased in p38γ-depleted cells, further supporting oxidative injury response (Fig. 6O). As expected, the control shC+Cas9-C treatment did not affect mitochondrial functions in NPC cells (Fig. 6M–O).

A recent study by Wang et al. has shown that p38γ is also essential for aerobic glycolysis and pancreatic tumorigenesis through PFKFB3 and GLUT2. p38γ KO reduced PFKFB3 phosphorylation, PFKFB3 and GLUT2 protein expression, and inhibited aerobic glycolysis to impede pancreatic cancer cell growth [12]. Here we found that p-PFKFB3/PFKFB3/GLUT2 expression was robustly decreased in p38γ-silenced or p38γ-KO CNE-1 cells (Fig. 6P), where the cellular ATP contents were decreased (Fig. 6Q). Together, these results showed that p38γ depletion disrupted mitochondrial functions and depleted ATP in NPC cells.

### p38γ silencing inhibits NPC xenograft tumor growth in nude mice

**Fig. 7:**
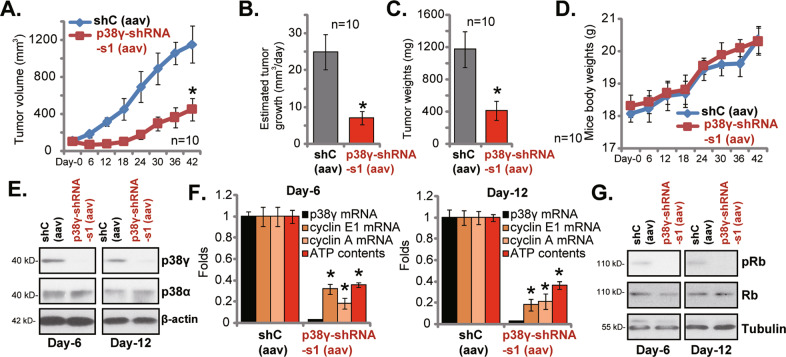
p38γ silencing inhibits NPC xenograft tumor growth in nude mice. The pNPC-1 xenograft -bearing nude mice were subject to intratumoral injection of aav-packed p38γ-shRNA [“p38γ-shRNA-s1 (aav)”] or aav-packed scramble control shRNA [“shC (aav)”]. Virus injection was performed daily for 12 consecutive days. The tumor growth curve results (**A**) and mice body weights (**D**) were recorded every six days. The estimated daily tumor growth, in mm^3^ per day, was calculated by the described formula (**B**). At Day-42, pNPC-1 xenografts were isolated and weighted individually (**C**). At Day-6 and Day-12, four hours after the virus injection, one tumor of each group was isolated. Expression of listed proteins (**E** and **G**), mRNAs (**F**) and ATP contents (**F**) in fresh tumor tissue lysates were shown (**E**). *P < 0.05 *vs*. “shC (aav)” group.

To examine the potential effect of *p38γ* on NPC cell growth in vivo, pNPC-1 primary cells (at 6 × 10^6^ cells per mouse) were *s.c*. injected to the flanks of the nude mice. NPC xenograft tumors were established within three weeks after cell injection (“Day-0”). The xenograft-bearing mice were then randomly assigned into two groups. The treatment group ten mice (*n* = 10) received intratumoral injection of aav-packed p38γ-shRNA [“p38γ-shRNA-s1 (aav)”]. The control group contained ten mice as well and subject to intratumoral injection of aav-packed scramble control shRNA [“shC (aav)”]. The intratumoral injection of the virus was performed daily for 12 consecutive days. The tumor growth curve results, in Fig. 7A, demonstrated that p38γ-shRNA-s1 (aav) injection robustly inhibited pNPC-1 xenograft growth in nude mice (Fig. 7A). The estimated daily tumor growth was calculated by the described formulation:(tumor volume at Day-42 subtracting tumor volume at Day-0)/42 [21], and results further showed that injection of p38γ-shRNA-s1 (aav) largely inhibited pNPC-1 xenograft growth in mice (Fig. 7B). At Day-42, pNPC-1 xenografts of the two group mice were isolated and weighted individually. Results in Fig. 7C demonstrated that pNPC-1 xenografts with p38γ-shRNA-s1 (aav) injection were significantly lighter than those with shC (aav) injection. The mice body weights, as shown in Fig. 7D, were not significantly different between the two groups.

At Day-6 and Day-12, four hours after the virus injection, one tumor of each group was isolated. Fresh tumor tissues were obtained from the four total tumors. As shown p38γ protein (Fig. 7E) and p*38γ* mRNA (Fig. 7F) were indeed silenced in the p38γ-shRNA-s1 (aav)-injected pNPC-1 xenograft tissues. p38α protein expression was unchanged (Fig. 7E). In line with the in vitro findings, we found that Rb phosphorylation (Fig. 7G), *cyclin E1* and *cyclin A* mRNA expression (Fig. 7F) and ATP levels (Fig. 7F) were dramatically decreased in p38γ-shRNA-expressing pNPC-1 xenograft tissues.

## Discussion

Recent studies have shown that p38γ can exert pro-tumorigenic activity in different human cancers. Wang et al. have shown that p38γ is overexpressed in pancreatic cancer, regulating KRAS oncogene signaling and aerobic glycolysis to promote tumorigenesis and cancer progression through PFKFB3-GLUT2 signaling [12]. Chen et al. have shown that p38γ overexpression-promoted renal cell carcinoma (RCC) cell growth, proliferation and migration, and it is a promising therapeutic target of RCC [14]. Su et al. found that p38γ expression is significantly elevated in colorectal cancer (CRC). p38γ silencing or knockout inhibited CRC cell growth, proliferation, and migration, and induced apoptosis activation [13]. In addition, Shi et al. reported that p38γ overexpression-promoted Rb phosphorylation and cyclin E1/cyclin A expression as well as osteosarcoma cell growth [22].

Here we provided strong evidence to support that p38γ is an important cancer-promoting gene and therapeutic target of NPC. GEPIA database and results from the local NPC tissues demonstrate that p38γ is significantly upregulated in NPC tissues, correlating with poor overall survival. Furthermore, *p38γ* mRNA and protein expression is significantly elevated in established and primary human NPC cells, whereas low expression detected in nasal epithelial cells. In NPC cells, p38γ shRNA or CRISPR/Cas9-induced p38γ KO potently inhibited cell growth, proliferation, migration and invasion, and induced significant apoptosis activation. Contrarily, ectopic overexpression of p38γ exerted opposite activity and promoted NPC cell proliferation and migration. Importantly, p38γ shRNA failed to affect viability and proliferation in nasal epithelial cells. In vivo, intratumoral injection of p38γ shRNA aav potently inhibited primary human NPC xenograft growth in nude mice. Therefore, targeting p38γ could be a novel strategy to inhibit NPC.

Studies have shown that CDK-Rb-cyclin is dysregulated in NPC. Roniciclib (BAY1000394), a potent pan-CDK inhibitor, displayed promising anti-cancer activity in preclinical NPC models, either alone or in combination with cisplatin [32]. Niu et al. found that C-myc silencing inhibited NPC cell proliferation by inhibiting CDK-Rb-cyclin pathway [33]. Wu et al. reported that microRNA-188 (miR-188) exerted anti-cancer activity by targeting the CDK-Rb-E2F cascade in NPC cells [34].

Recent studies have proposed p38γ as a non-classical CDK-like kinase, phosphorylating and inhibiting the tumor suppressor protein Rb to promote the expression of cyclin A and cyclin E1. This will lead to cell cycle progression and cancer cell growth [14, 20]. In line with these findings, we found that Rb phosphorylation as well as cyclin E1/A expression were significantly decreased in p38γ-silenced or p38γ-KO NPC cells, but increased after p38γ overexpression. Moreover, Rb phosphorylation and cyclin E1/A expression were decreased in pNPC-1 xenograft tumors with p38γ shRNA aav injection. The non-phosphorylated Rb-mut blocked Rb phosphorylation and decreased cyclin E1/A expression in OE-p38γ-L1 CNE-1 cells. The p38γ specific inhibitor PFD inhibited Rb phosphorylation and cyclins expression in NPC cells. Thus, p38γ-driven NPC progression is, at least in part, due to regulating Rb-cyclin cascade.

Increased mitochondrial function is essential for the progression of NPC [35–37]. Several key mitochondrial components were upregulated and/or hyper-activated in NPC, associated with tumorigenesis and cancer progression [35–37]. Conversely, mitochondria damage or dysregulation can induce death of NPC cells [35–37]. One important finding of this study is that p38γ localizes in mitochondria in NPC cells. p38γ was important for mitochondrial functions. On the contrary, p38γ shRNA or KO disrupted mitochondrial functions, causing mitochondrial depolarization, ROS production, oxidative injury and ATP depletion in NPC cells. In vivo, ATP depletion was detected in p38γ shRNA aav-injected pNPC-1 xenograft tissues. Therefore, maintaining mitochondrial function could be another mechanism of p38γ-driven NPC progression.

## Conclusion

The current clinical treatments for the advanced NPC is still challenging and it is therefore extremely important to uncover the novel therapeutic targets for NPC. The results of this study suggest that p38γ is a key oncogenic gene and an important therapeutic target of NPC.

## Supplementary information

Figure S1.

aj-checklist

Author contribution form

## Data Availability

All data are available upon request.
