# Supplementary figures and images for: The pro-tumorigenic activity of p38γ overexpression in nasopharyngeal carcinoma
Source: Cell Death Dis. 2022 Mar 4;13(3):210. doi: 10.1038/s41419-022-04637-8 (PMC8897421; doi:10.1038/s41419-022-04637-8)

Figure S1. The uncropped blotting images

Figure 1

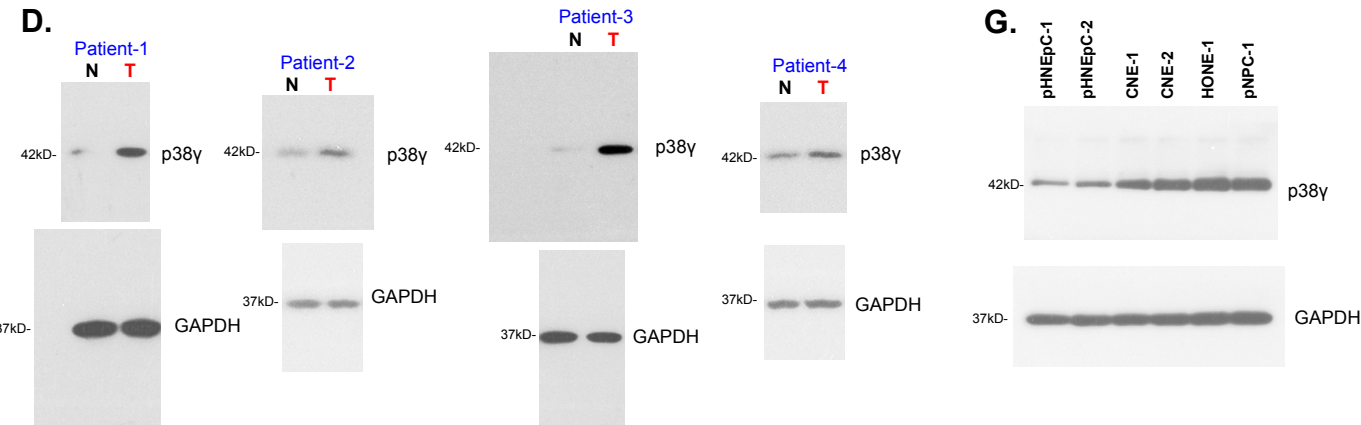

Figure 4

Figure 2

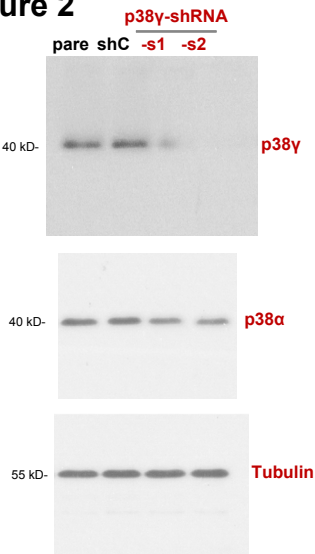

C.

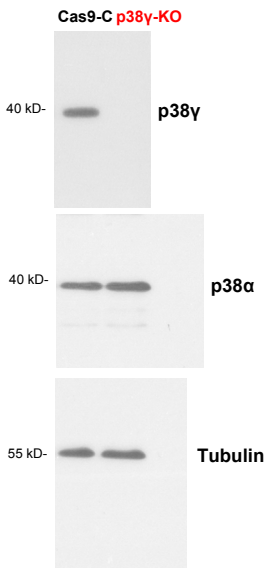

G.

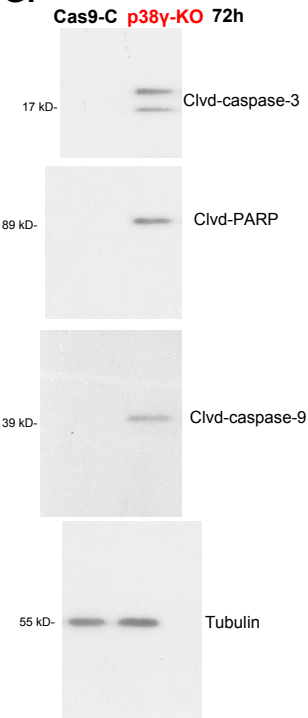

Figure 5

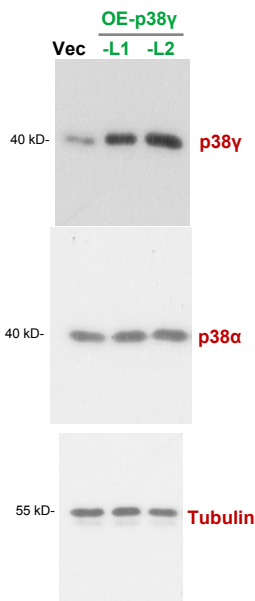

Figure 6

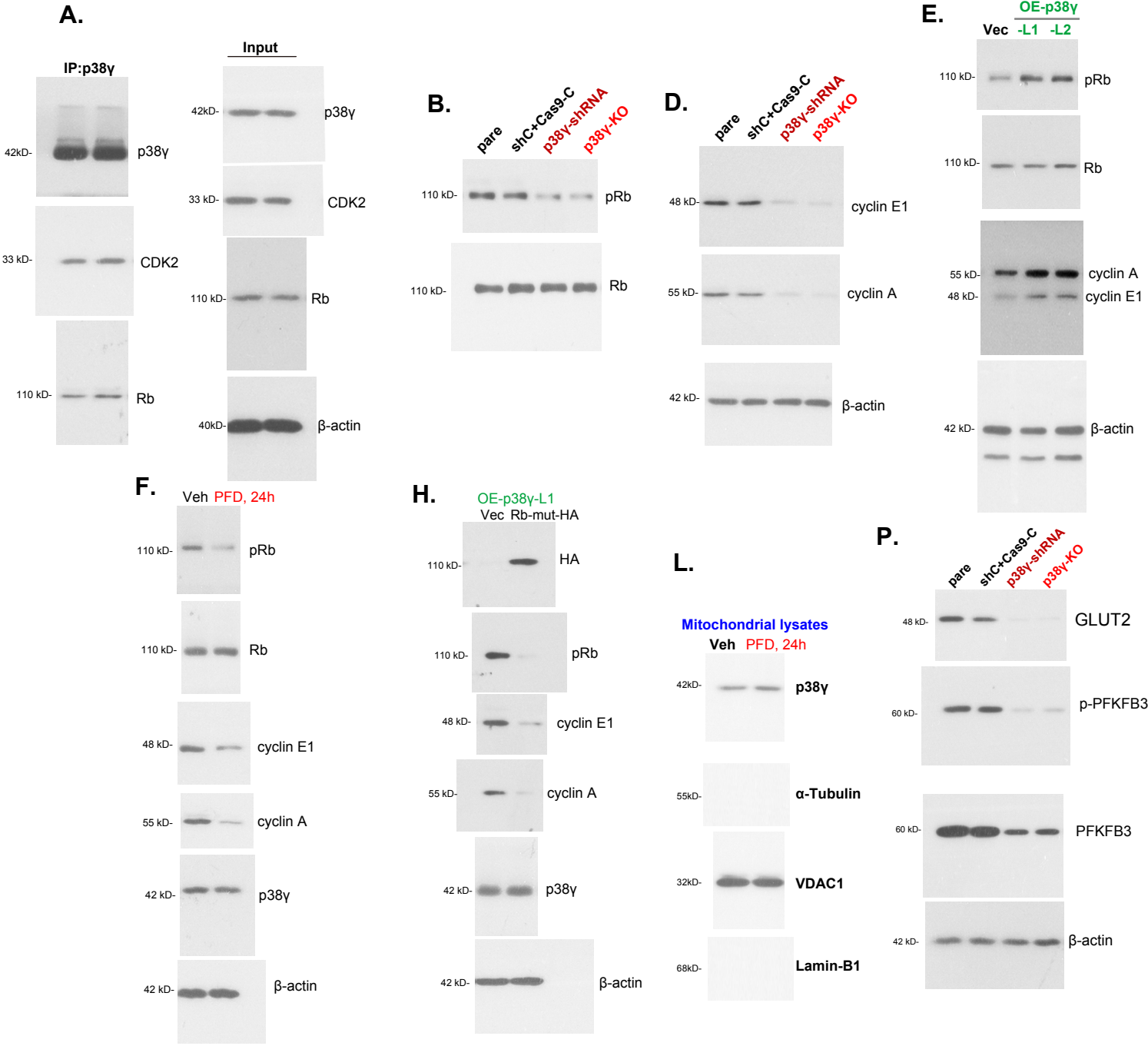

Figure 7

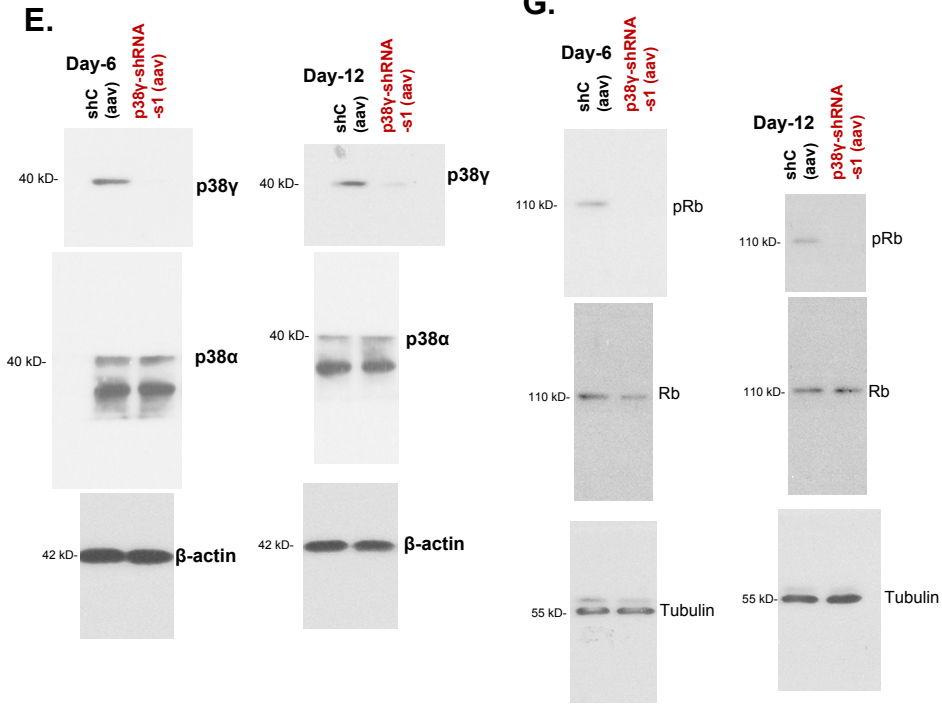

Supplement: Supplementary file 1 — Figure S1. [file 41419_2022_4637_MOESM1_ESM.pdf]
