# Supplementary material for: The pro-tumorigenic activity of p38γ overexpression in nasopharyngeal carcinoma
Source: Cell Death Dis. 2022 Mar 4;13(3):210. doi: 10.1038/s41419-022-04637-8 (PMC8897421; doi:10.1038/s41419-022-04637-8)
Supplement: Supplementary file 3 — Author contribution form [file 41419_2022_4637_MOESM3_ESM.pdf]

DECLARATION OF CONTRIBUTIONS TO ARTICLE

ADMC

Manuscript Number:

Journal Name: *Cell Death & Disease* (the 'Journal')

Proposed Title of the Contribution:

The pro-tumorigenic activity of p38γ overexpression in nasopharyngeal carcinoma (the 'Contribution)

Author(s):

De-Pei Yin, Peng Sun, Ming-Yu Yao, Yu-Fan Zheng, Li-xiao Xie, Xun-Wu Dou, Ye Tian, Ji-Sheng Liu (the 'Authors')

For all *CDDis* articles, each person named as an author in the published version must be able to show he or she has contributed substantially to the article.

Authorship credit should be based on 1) substantial contributions to conception and design, acquisition of data, or analysis and interpretation of data; 2) drafting the article or revising it critically for important intellectual content; and 3) final approval of the version to be published. Authors should meet conditions 1, 2 and 3.

Any person who cannot be shown to have made a substantial contribution to the article cannot be listed as an author in the final version. The name of any person who is deemed to have made a minor contribution can, however, appear in the Acknowledgments section of the article.

Please complete the table below to indicate the contributions of all named authors to the manuscript.

Author Full Name:	Specification of Contribution to the Manuscript:
De-Pei Yin	Functional assays in NPC cells and normal cells, establishing NPC xenografts, testing signalings and cell death/apoptosis, drafting the article and revising it, with final approval of the version submitted to the journal.
Peng Sun	Functional assays in NPC cells and normal cells, establishing NPC xenografts, testing signalings and cell death/apoptosis, drafting the article and revising it, with final approval of the version submitted to the journal.
Ming-Yu Yao	Functional assays in NPC cells and normal cells, establishing NPC xenografts, testing signalings and cell death/apoptosis, drafting the article and revising it, with final approval of the version submitted to the journal.
Yu-Fan Zheng	Testing signaling in NPC cancer tissues, drafting the article and revising it, with final approval of the version submitted to the journal.
Li-xiao Xie	Establishing NPC xenografts, Figure organization, involved in drafting the article and revising it critically for important intellectual content, and with final approval of the version submitted to the journal.
Xun-Wu Dou	Study conception and design, and data analysis, Figure organization, involved in drafting the article and revising it critically for important intellectual content, and with final approval of the version submitted to the journal.
Ye Tian	Study conception and design, and data analysis, Figure organization, involved in drafting the article and revising it critically for important intellectual content, and with final approval of the version submitted to the journal.
Ji-Sheng Liu	Study conception and design, and data analysis, Figure organization, involved in drafting the article and revising it critically for important intellectual content, and with final approval of the version submitted to the journal.

Please complete the table below to indicate the contributions of all named authors to the figures.

Figure 1:

De-Pei Yin, Peng Sun, Ming-Yu Yao, Yu-Fan Zheng, Xun-Wu Dou, Ye Tian and Ji-Sheng Liu

Figure 2:

De-Pei Yin, Peng Sun, Ming-Yu Yao, Xun-Wu Dou, Ye Tian and Ji-Sheng Liu

Figure 3:

De-Pei Yin, Ming-Yu Yao, Yu-Fan Zheng, Li-xiao Xie, Xun-Wu Dou, Ye Tian and Ji-Sheng Liu

Figure 4:

De-Pei Yin, Peng Sun, Ming-Yu Yao, Yu-Fan Zheng, Li-xiao Xie, Xun-Wu Dou, Ye Tian and Ji-Sheng Liu

Figure 5:

De-Pei Yin, Peng Sun, Ming-Yu Yao, Yu-Fan Zheng, Li-xiao Xie, Xun-Wu Dou, Ye Tian and Ji-Sheng Liu

Figure 6:

De-Pei Yin, Peng Sun, Ming-Yu Yao, Yu-Fan Zheng, Li-xiao Xie, Xun-Wu Dou, Ye Tian and Ji-Sheng Liu

Figure 7

De-Pei Yin, Peng Sun, Ming-Yu Yao, Yu-Fan Zheng, Li-xiao Xie, Xun-Wu Dou, Ye Tian and Ji-Sheng Liu

Signed for and on behalf of the Author(s):	Print Name:	Date:
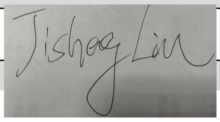	Ji-Sheng Liu	11-04-2021
